# Supplementary material for: Integrated Surveillance of Disparities in Vaccination Coverage and Morbidity during the COVID-19 Pandemic: A Cohort Study in Southeast Sweden
Source: Vaccines (Basel). 2024 Jul 12;12(7):763. doi: 10.3390/vaccines12070763 (PMC11281347; doi:10.3390/vaccines12070763)
Supplement: Supplementary file 1 [file vaccines-12-00763-s001.zip › vaccines-3073046-supplementary.pdf]

Supplementary materials

Supplementary Figures

Figure S1-S9. p. 2–10

Directed Acyclic Graphs (DAGs) of associations between exposures and study outcomes.

Supplementary Text

Text S1. p. 11

Detailed outline of sensitivity analysis.

Supplementary Tables

Table S1. p. 12–16

Distribution (n, %) of Birth Countries of Western Born and Persons with Relocated Status

Table S2. p. 17

Study Population Displayed by Age Group and Relocation Status. Distribution (n, %) of Birth Countries of Western Born and Persons with Relocated Status

Table S3. p. 18

Distribution (n, %) of Substance Abuse and Psychotic Diagnoses in Western Born and Persons with Relocated Status

Table S4. p. 19

Likelihood of Remaining Unvaccinated among Adult Residents and Psychiatric Populations in Östergötland and Jönköping Counties, Sweden, February 2020 to February 2022 – Simple models

Table S5. p. 20

Likelihoods of COVID-19 Monitoring by Proactive Testing and Testing Positive for SARS-COV-2 among Adult Residents and Psychiatric Populations in Östergötland and Jönköping Counties, Sweden, February 2020 to February 2022 – Simple models

Table S6. p. 21

Likelihood of Hospitalization With COVID-19 among Adult Residents and Psychiatric Populations in Östergötland and Jönköping Counties, Sweden, February 2020 to February 2022 – Simple models

Table S7. p. 22

Likelihood of Hospitalization with COVID-19 in Vaccination Period in Patients Diagnosed with Substance Abuse or a Psychotic Disorder Displayed by Vaccination Status – Simple models.

Directed acyclical graphs (DAGs) of assumed associations between exposures and study outcomes

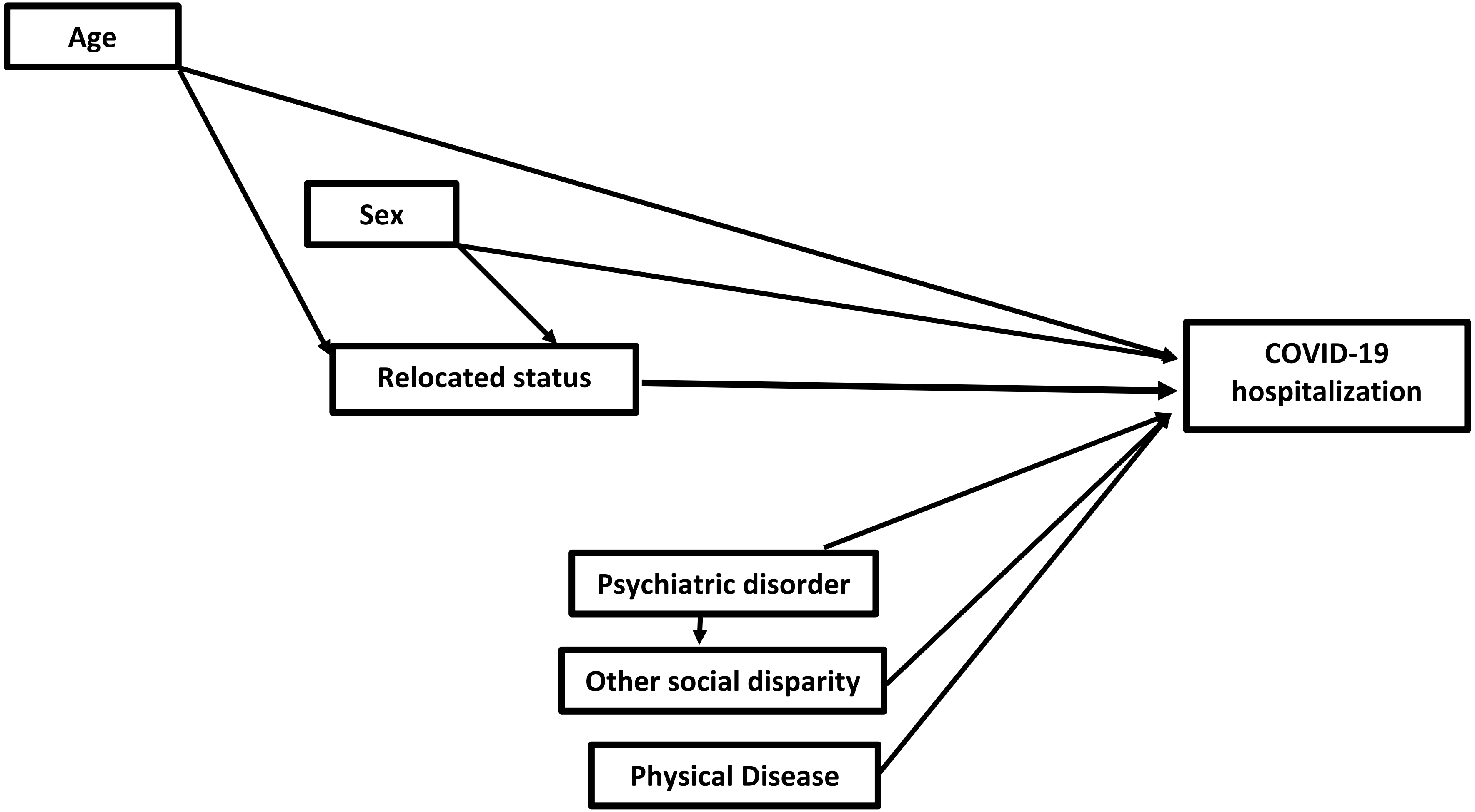

Figure S1. Assumed association between main exposure relocated status and primary outcome COVID-19 hospitalization.

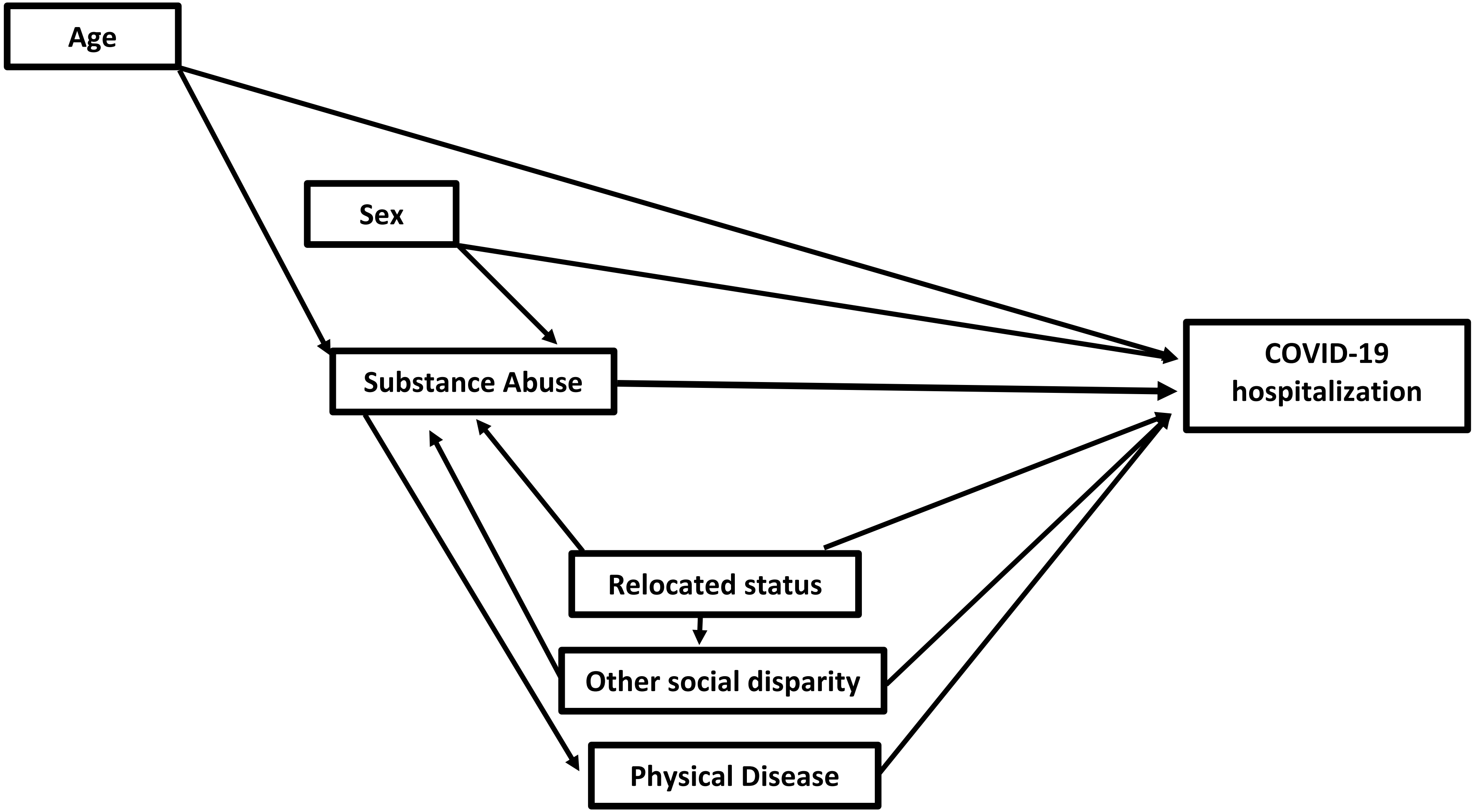

Figure S2. Assumed association between main exposure substance abuse and primary outcome COVID-19 hospitalization.

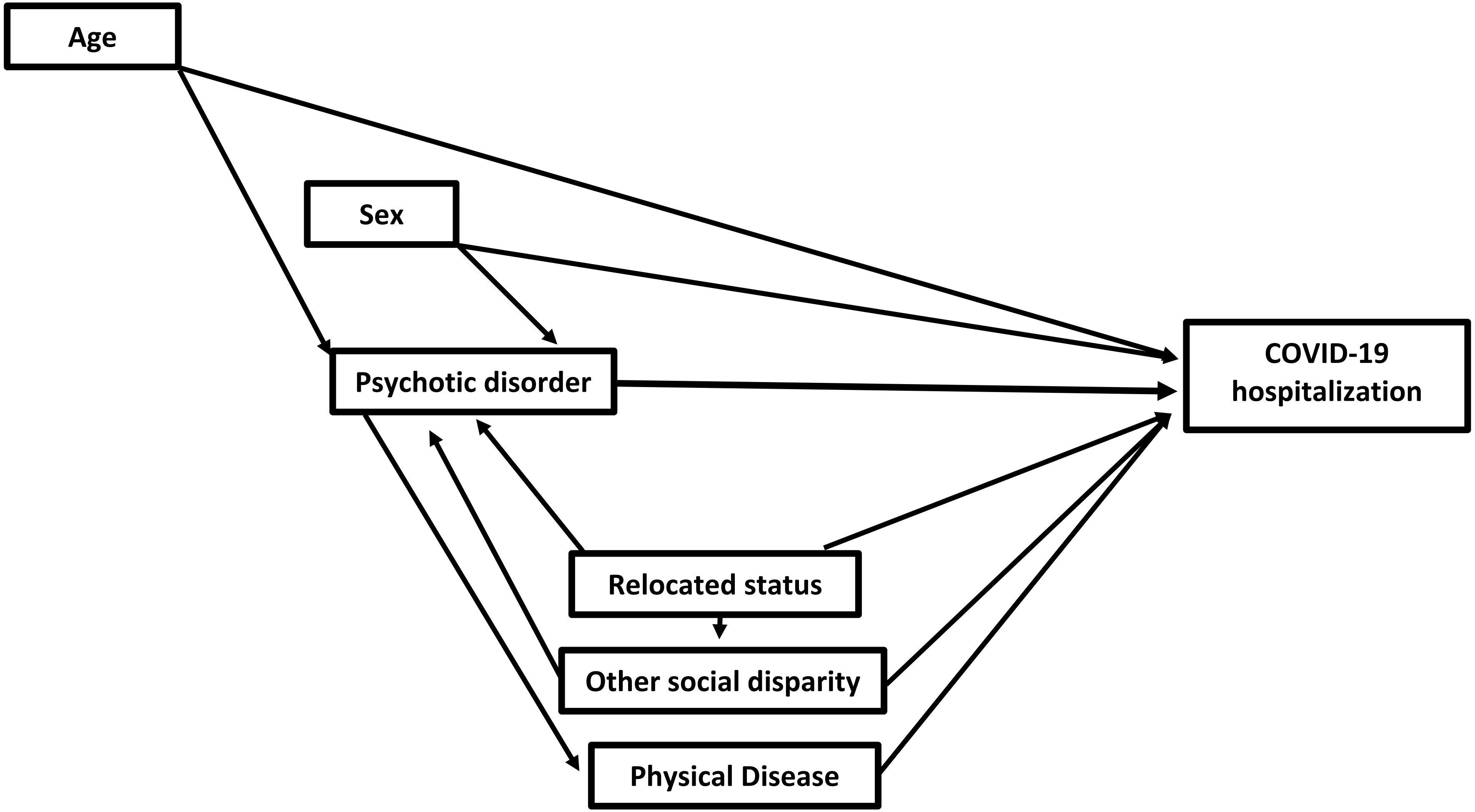

Figure S3. Assumed association between main exposure psychotic disorder and primary outcome COVID-19 hospitalization.

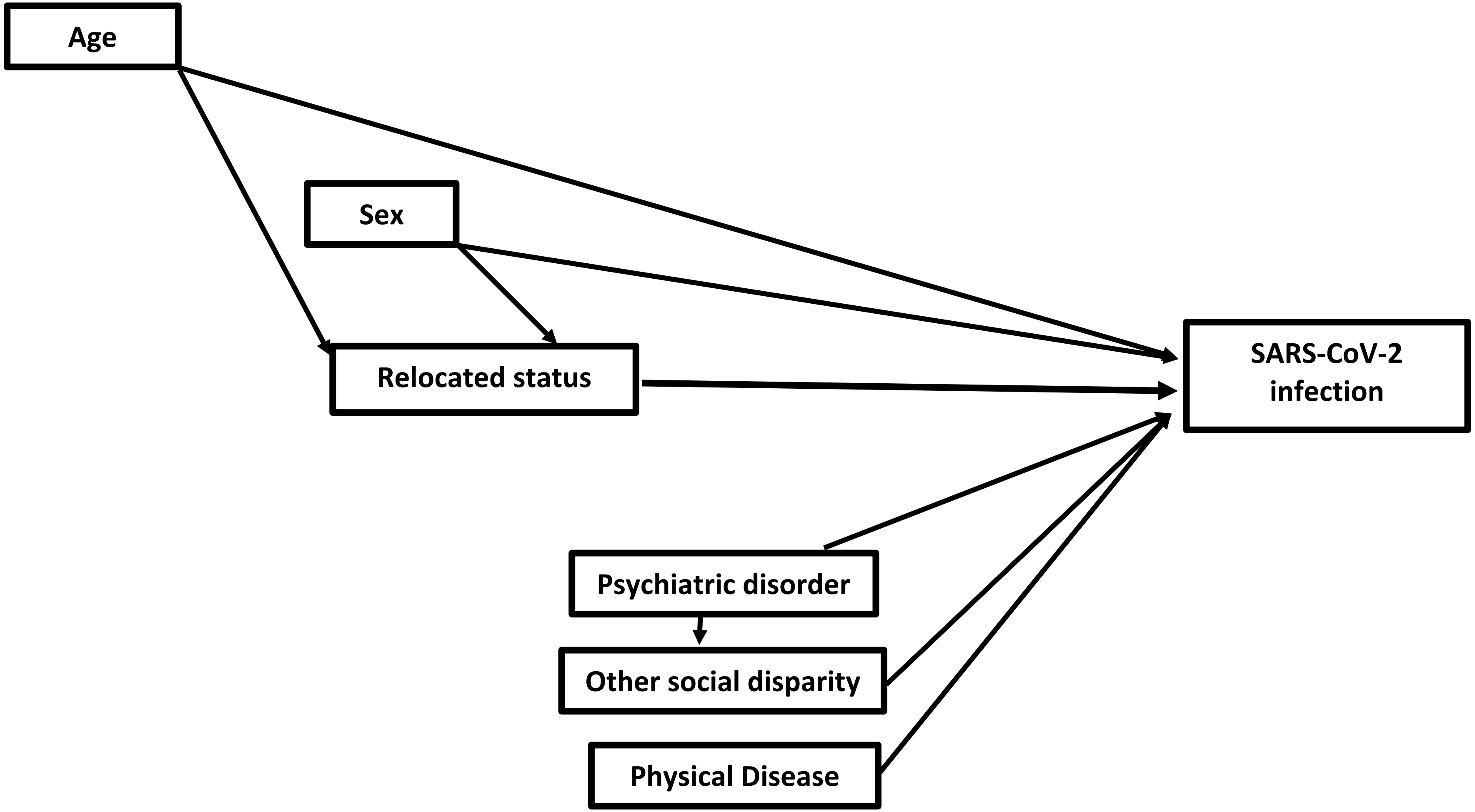

Figure S4. Assumed association between main exposure relocated status and secondary outcome SARS-CoV-2 infection.

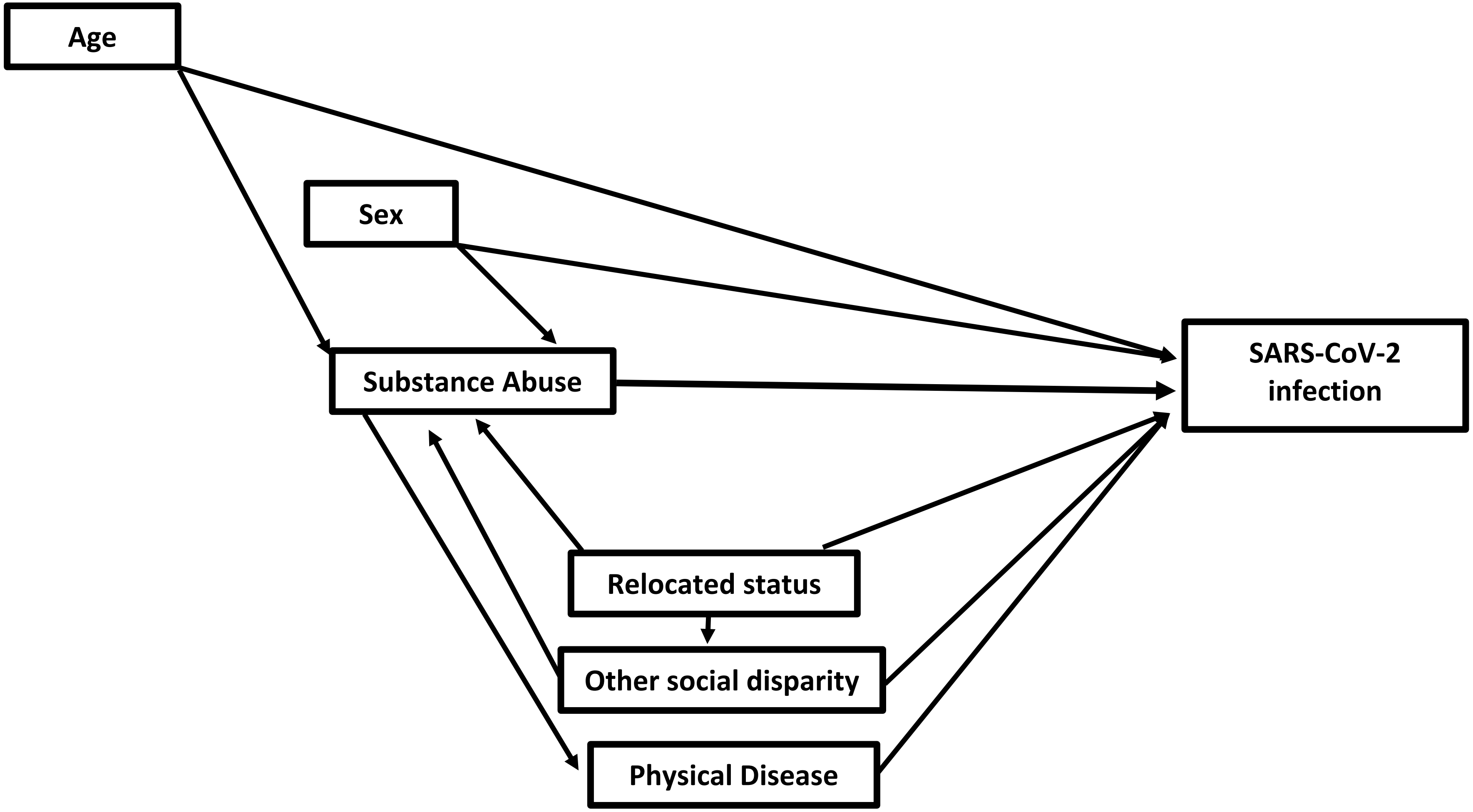

Figure S5. Association between main exposure substance abuse and SARS-CoV-2 infection.

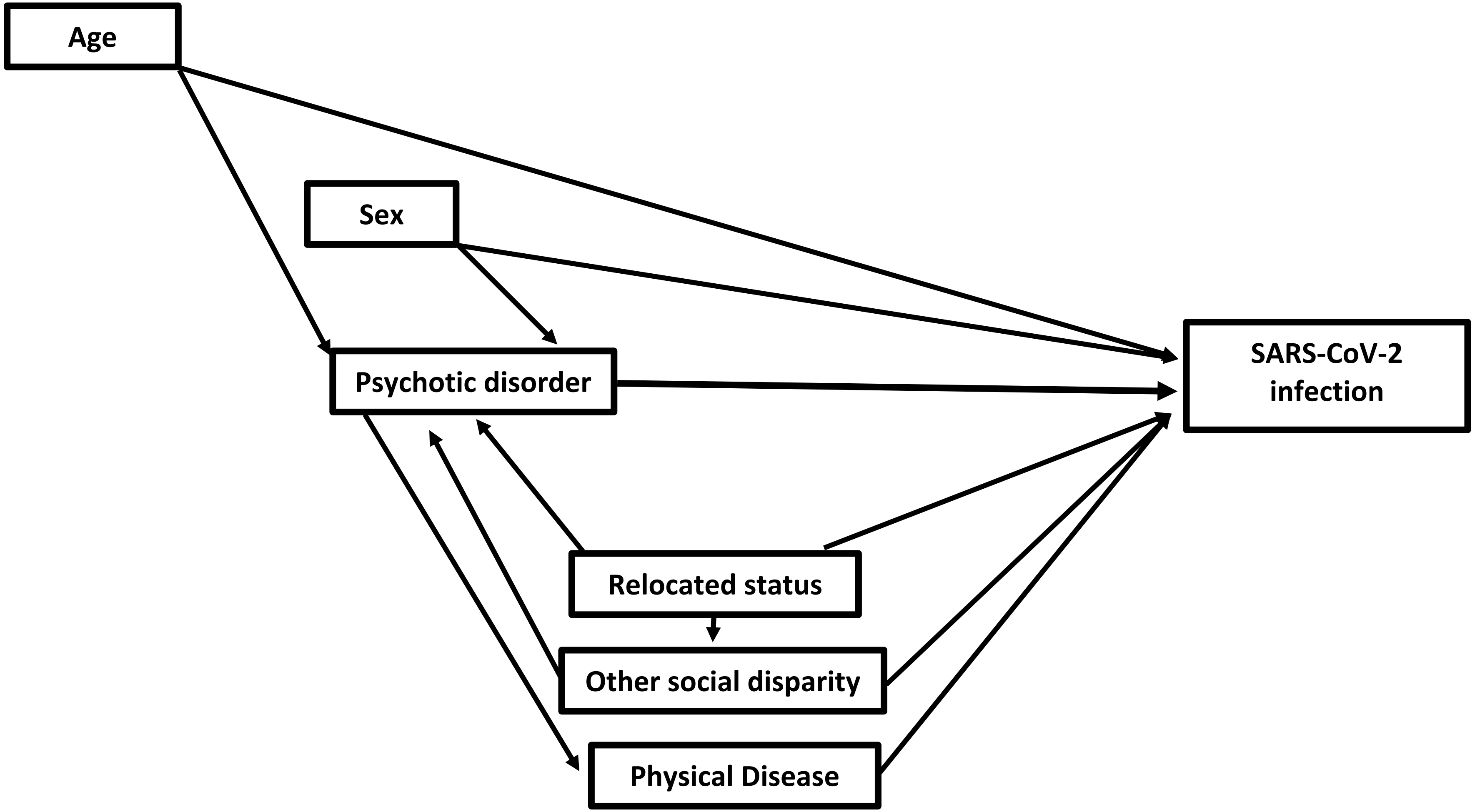

Figure S6. Association between main exposure psychotic disorder and SARS-CoV-2 infection.

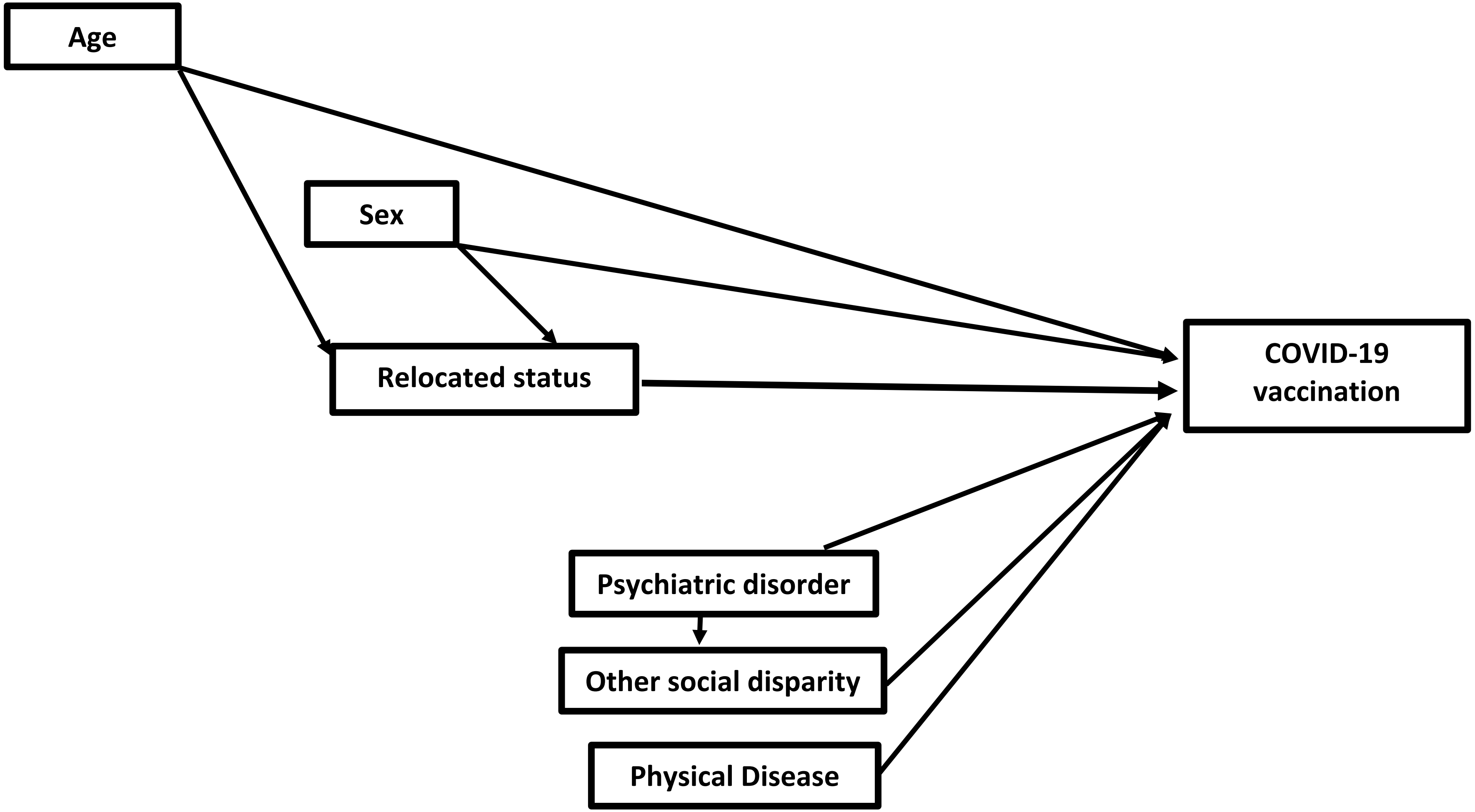

Figure S7. Assumed association between main exposure relocated status and secondary outcome COVID-19 vaccination.

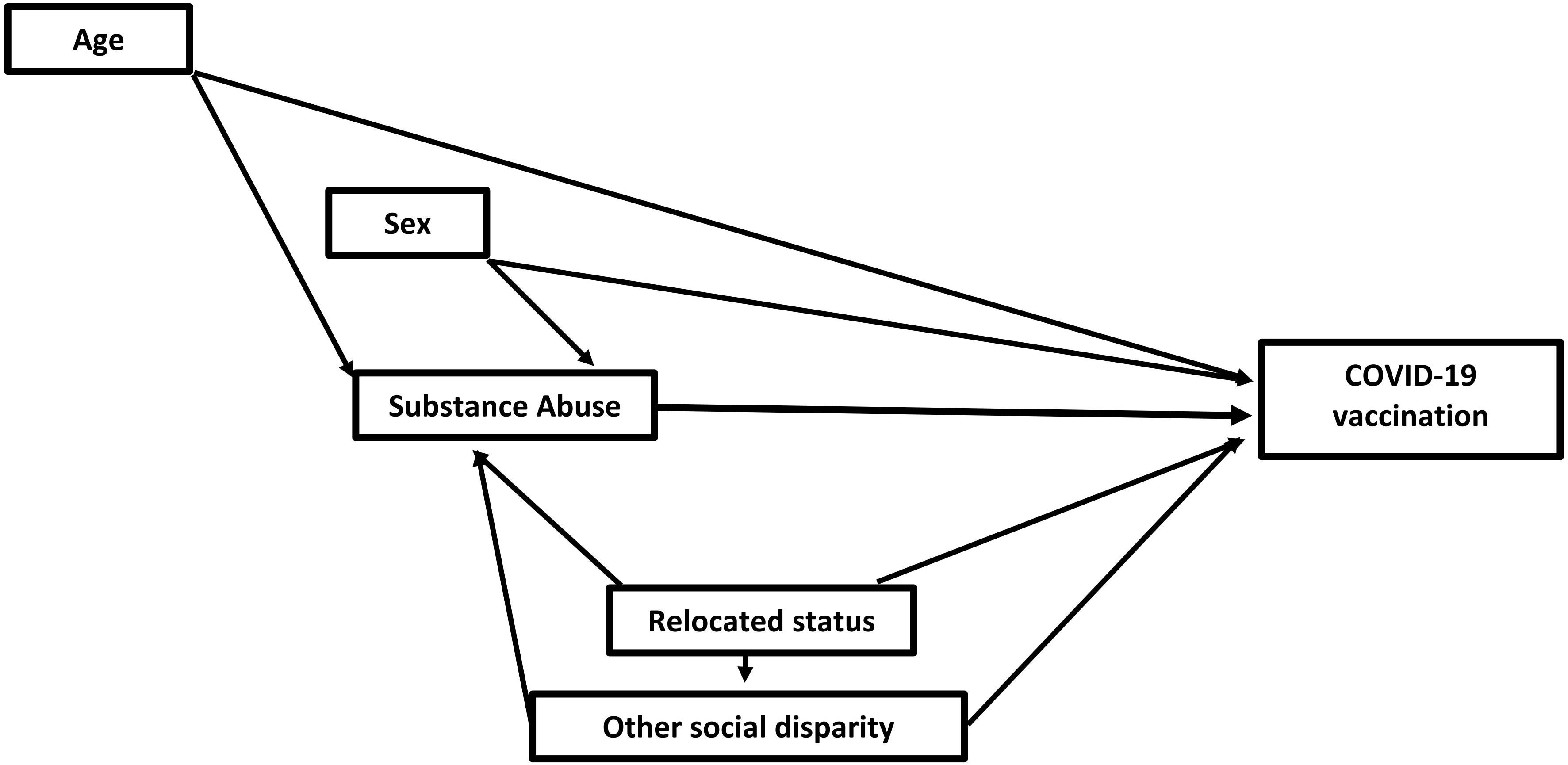

Figure S8. Association between main exposure substance abuse and COVID-19 vaccination.

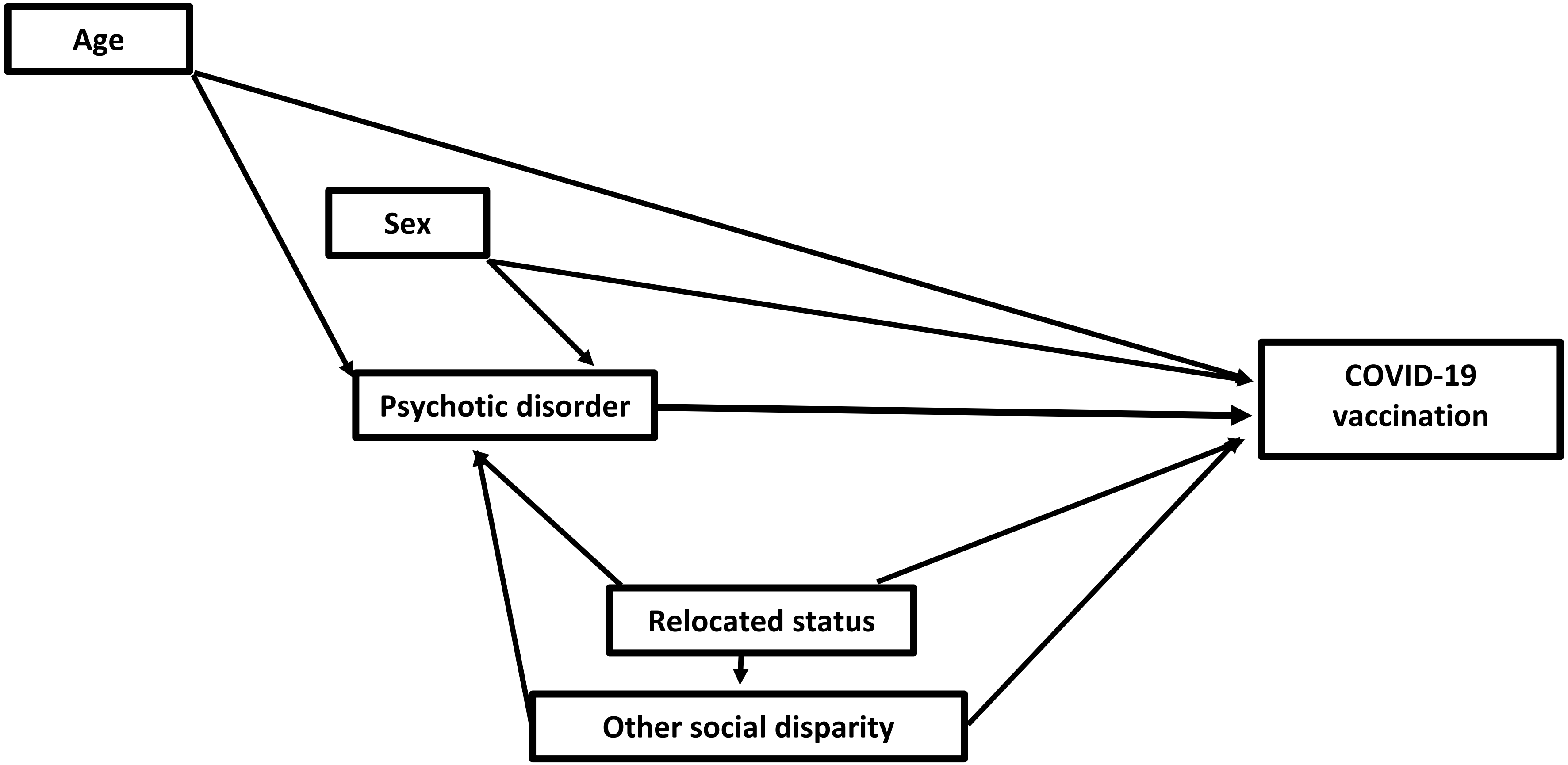

Figure S9. Association between main exposure psychotic disorder and COVID-19 vaccination.

Supplementary Text

Text S1. Detailed outline of sensitivity analysis.

The E value quantifies the minimum strength of association on the rate ratio (RR) scale that a hypothetical unmeasured confounder would need to have with both the outcome and the exposure to explain away the estimated exposure–outcome association.[11] The confidence limit of the E value closest to the null is of higher interest than the E value of the point estimate. This confidence limit is calculated by replacing the RR by the lower or the upper confidence limit of the RR, depending on which of the two are closest to the null (if RR > 1, the lower confidence limit is closest to the null; and if RR < 1, the upper confidence limit is closest to the null). Because the outcome measure used in this study is the odds ratio (OR) instead of the RR, the E value was calculated differently depending on whether the outcome was rare (prevalence approximately <15% or >85%) or was not rare (prevalence approximately between 15% and 85%).

If the outcome was rare (<15%), the OR approximates the RR and the original E value formulas were used (LL = Lower limit, UL = Upper limit):

(1) If OR > 1: $E\ value\ (Point\ estimate) = OR + \sqrt{OR * (OR - 1)}$

$$E\ value\ (LL) = 1\ if\ LL \leq 1,\ else\ LL + \sqrt{LL * (LL - 1)}.$$

If OR < 1: $E\ value\ (Point\ estimate) = 1/OR + \sqrt{1/OR * (1/OR - 1)}$

$$E\ value\ (UL) = 1\ if\ UL \geq 1,\ else\ 1/UL * \sqrt{1/UL * (1/UL - 1)}.$$

Else an approximate E value was obtained by replacing the RR with the square root of the OR:

(2) If OR > 1: $E\ value\ (Point\ estimate) = \sqrt{OR} + \sqrt{\sqrt{OR} * \left(\sqrt{OR} - 1\right)}$

$$E\ value\ (LL) = 1\ if\ LL \leq 1, else\ 1/\sqrt{LL} + \sqrt{1/\sqrt{LL} * \left(1/\sqrt{LL} - 1\right)}.$$

If OR < 1: $E\ value\ (Point\ estimate) = 1/\sqrt{OR} + \sqrt{1/\sqrt{OR} * \left(1/\sqrt{OR} - 1\right)}.$

$$E\ value\ (UL) = 1\ if\ UL \geq 1, else\ 1/\sqrt{UL} + \sqrt{1/\sqrt{UL} * \left(1/\sqrt{UL} - 1\right)}.$$

Higher E values correspond to situations in which the existence of an unmeasured confounder able to tip the estimated exposure–outcome relationship is more unlikely.

Reference

30. VanderWeele TJ, Ding P. Sensitivity analysis in observational research: introducing the E-value. *Ann Intern Med* 2017; **167**(4): 268–274. doi:10.7326/M16-–2607

Supplementary tables

Table S1. Distribution (n, %) of Birth Countries of Western Born and Persons with Relocated Status.

**Western born**	**n**	**% western born**	**% all**
Sweden	527,249	96.3	80.1
Finland	7319	1.3	1.1
Germany	3692	0.7	0.6
Denmark	1664	0.3	0.3
Norway	1476	0.3	0.2
United Kingdom	1252	0.2	0.2
USA	1016	0.2	0.2
Netherlands	808	0.1	0.1
Spain	551	0.1	0.1
Italy	511	0.1	0.1
France	384	0.1	0.1
Austria	330	0.1	0.1
Iceland	251	0.0	0.0
Switzerland	195	0.0	0.0
Australia	179	0.0	0.0
Canada	184	0.0	0.0
Portugal	148	0.0	0.0
Belgium	113	0.0	0.0
Ireland	81	0.0	0.0
New Zealand	56	0.0	0.0
Luxembourg	8	0.0	0.0
Malta	8	0.0	0.0
Total	547,475	100	83.2

**Relocated status**	**n**	**% relocated status**	**% all**
Syria	18,345	16.6	2.8
Iraq	11,581	10.5	1.8
Bosnia Herzegovina	9108	8.2	1.4
Somalia	6211	5.6	0.9
Yugoslavia	5731	5.2	0.9
Poland	4220	3.8	0.6
Afghanistan	4197	3.8	0.6
Iran	3608	3.3	0.5
Thailand	2791	2.5	0.4
Eritrea	2569	2.3	0.4
Turkey	2397	2.2	0.4
Romania	2359	2.1	0.4
India	2180	2.0	0.3
Vietnam	2113	1.9	0.3
Lebanon	1923	1.7	0.3
Chile	1776	1.6	0.3
China	1732	1.6	0.3

Croatia	1318	1.2	0.2
Russia	1186	1.1	0.2
Pakistan	1083	1.0	0.2
Serbia	1051	1.0	0.2
Lithuania	982	0.9	0.1
Hungary	963	0.9	0.1
Philippines	911	0.8	0.1
Greece	876	0.8	0.1
Kosovo	823	0.7	0.1
Ethiopia	822	0.7	0.1
Serbia and Montenegro	741	0.7	0.1
Colombia	724	0.7	0.1
South Korea	688	0.6	0.1
Republic of the Congo	617	0.6	0.1
Brazil	563	0.5	0.1
Sri Lanka	547	0.5	0.1
Ukraine	526	0.5	0.1
North Macedonia	525	0.5	0.1
Bulgaria	518	0.5	0.1
Latvia	503	0.5	0.1
Burundi	453	0.4	0.1
Estonia	453	0.4	0.1
Morocco	412	0.4	0.1
Albania	407	0.4	0.1
Saudi Arabia	402	0.4	0.1
Sudan	392	0.4	0.1
Palestine (including Gaza and the West Bank)	390	0.4	0.1
Bangladesh	340	0.3	0.1
Czechoslovakia	325	0.3	0.0
Egypt	293	0.3	0.0
Kuwait	272	0.2	0.0
Nigeria	262	0.2	0.0
Uganda	256	0.2	0.0
Uzbekistan	246	0.2	0.0
Cameroon	234	0.2	0.0
Libya	230	0.2	0.0
Yemen	226	0.2	0.0
Armenia	223	0.2	0.0
Jordan	220	0.2	0.0
Kenya	217	0.2	0.0
Tunisia	208	0.2	0.0
Peru	198	0.2	0.0
Azerbaijan	178	0.2	0.0
Belarus	177	0.2	0.0
United Arab Emirates	175	0.2	0.0
The Gambia	173	0.2	0.0

Ghana	172	0.2	0.0
Mexico	170	0.2	0.0
South Africa	159	0.1	0.0
Japan	146	0.1	0.0
The Democratic Republic of Kongo	145	0.1	0.0
Indonesia	143	0.1	0.0
Czech Republic	141	0.1	0.0
Israel	135	0.1	0.0
Tanzania	134	0.1	0.0
Argentina	131	0.1	0.0
Bolivia	123	0.1	0.0
Myanmar	123	0.1	0.0
El Salvador	121	0.1	0.0
Slovakia	117	0.1	0.0
Slovenia	116	0.1	0.0
Taiwan	101	0.1	0.0
Montenegro	97	0.1	0.0
Ecuador	90	0.1	0.0
Algeria	89	0.1	0.0
Kazakhstan	88	0.1	0.0
Moldova	84	0.1	0.0
Rwanda	84	0.1	0.0
Mongolia	78	0.1	0.0
Liberia	73	0.1	0.0
Malaysia	70	0.1	0.0
Venezuela	70	0.1	0.0
Cambodia	63	0.1	0.0
Cuba	63	0.1	0.0
Soviet Union	61	0.1	0.0
Zimbabwe	60	0.1	0.0
Guatemala	57	0.1	0.0
Cyprus	53	0.0	0.0
Kyrgyzstan	52	0.0	0.0
Georgia	51	0.0	0.0
Uruguay	50	0.0	0.0
Djibouti	45	0.0	0.0
Laos	41	0.0	0.0
Singapore	39	0.0	0.0
Nepal	38	0.0	0.0
Zambia	38	0.0	0.0
Angola	35	0.0	0.0
Sierra Leone	35	0.0	0.0
Costa Rica	33	0.0	0.0
Dominican Republic	32	0.0	0.0
Honduras	31	0.0	0.0
Ivory Coast	29	0.0	0.0

Nicaragua	28	0.0	0.0
Mozambique	25	0.0	0.0
Guinea	24	0.0	0.0
Togo	23	0.0	0.0
Hong Kong	20	0.0	0.0
Jamaica	19	0.0	0.0
Senegal	19	0.0	0.0
Panama	18	0.0	0.0
Qatar	17	0.0	0.0
Tajikistan	16	0.0	0.0
Paraguay	14	0.0	0.0
Central African Republic	12	0.0	0.0
Trinidad and Tobago	12	0.0	0.0
Bahrain	10	0.0	0.0
Cape Verde	10	0.0	0.0
South Sudan	10	0.0	0.0
Turkmenistan	7	0.0	0.0
Benin	6	0.0	0.0
Mali	6	0.0	0.0
Mauritius	6	0.0	0.0
North Korea	6	0.0	0.0
Barbados	5	0.0	0.0
Niger	5	0.0	0.0
Suriname	5	0.0	0.0
Botswana	4	0.0	0.0
Guyana	4	0.0	0.0
Namibia	4	0.0	0.0
Samoa	4	0.0	0.0
Burkina Faso	3	0.0	0.0
Gabon	3	0.0	0.0
Madagascar	3	0.0	0.0
Malawi	3	0.0	0.0
Swaziland	3	0.0	0.0
Fiji	2	0.0	0.0
Granada	2	0.0	0.0
Guinea-Bissau	2	0.0	0.0
Haiti	2	0.0	0.0
Mauritania	2	0.0	0.0
Papua New Guinea	2	0.0	0.0
Bahamas	1	0.0	0.0
Brunei	1	0.0	0.0
Dominica	1	0.0	0.0
Palau	1	0.0	0.0
Saint Lucia	1	0.0	0.0
Solomon Islands	1	0.0	0.0
Sao Tome and Principe	1	0.0	0.0

Seychelles	1	0.0	0.0
East Timor	1	0.0	0.0
Total	110,451	100	16.8

Table S2. Study Population Displayed by Age Group and Relocation Status.

Age (years)	Western born, *n* (%)	Relocated status, *n* (%)	All, *n* (%)
18–29	46,075 (7.0)	2077 (0.3)	48,152 (7.3)
30–49	118,034 (17.9)	8861 (1.3)	126,895 (19.3)
50–64	126,959 (19.3)	23,437 (3.6)	150,396 (22.9)
65–79	156,301 (23.8)	50,914 (7.7)	207,215 (31.5)
80+	100,106 (15.2)	25,219 (3.8)	125,268 (19.0)
Total	547,475 (83.2)	110,451 (16.8)	657,926 (100)

Table S3. Distribution (n, %) of Substance Abuse and Psychotic Diagnoses in Western Born and Persons with Relocated Status.

	Western born		Relocated status		Total	
	**n**	**%**	**n**	**%**	**n**	**%**
**Substance abuse**	**12,994**	**2.4**	**1268**	**1.1**	**14,262**	**2.2**
**F10** Alcohol-related disorders	9730	1.8	678	0.6	10,408	1.6
**F11** Opioid-related disorders	1266	0.2	196	0.2	1462	0.2
**F12** Cannabis-related disorders	788	0.1	217	0.2	1005	0.2
**F14** Cocaine-related disorders	70	0.0	11	0.0	81	0.0
**F15** Other stimulant-related disorders	587	0.1	55	0.0	642	0.1
**F16** Hallucinogen-related disorders	46	0.0	8	0.0	54	0.0
**F18** Inhalant-related disorders	26	0.0	4	0.0	30	0.0
**F19** Other psychoactive substance-related disorders	2658	0.5	350	0.3	3008	0.5
**Psychotic disorders**	**7202**	**1.3**	**1185**	**1.1**	**8387**	**1.3**
**F20** Schizophrenia	1265	0.2	308	0.3	1573	0.2
**F23** Brief psychotic disorder	808	0.1	258	0.2	1066	0.2
**F25** Schizoaffective disorders	579	0.1	142	0.1	721	0.1
**F28** Other psychotic disorder	47	0.0	19	0.0	66	0.0
**F29** Unspecified psychosis	858	0.2	292	0.3	1150	0.2
**F323** Major depressive disorder, single episode, severe with psychotic features	308	0.1	112	0.1	420	0.1
**F30** Manic episode	153	0.0	18	0.0	171	0.0
**F31** Bipolar disorder	4161	0.8	322	0.3	4483	0.7

Table S4. Likelihood of Remaining Unvaccinated among Adult Residents and Psychiatric Populations in Östergötland and Jönköping Counties, Sweden, February 2020 to February 2022 – Simple models.

	**Remaining unvaccinated, OR (95% CI) (*P* value)**
**Main exposures**	
Relocated (1, yes; 0, no)	3.04 (2.99–3.08) (<0.001)
Substance abuse (1, yes; 0, no)	1.68 (1.62–1.75) (<0.001)
Psychotic disorder (1, yes; 0, no)	1.59 (1.51–1.67) (<0.001)
**Moderators**	
Sex (1, male; 0, female)	1.07 (1.06–1.08) (<0.001)
Age (continuous)	0.98 (0.98–0.98) (<0.001)

Odds ratios (simple logistic regression models) represent associations between substance abuse and psychotic disorder, and having received less than two doses of COVID-19 vaccine during the pandemic. *P* values >0.05 are not significant.

Table S5. Likelihoods of COVID-19 Monitoring by Proactive Testing and Testing Positive for SARS-COV-2 among Adult Residents and Psychiatric Populations in Östergötland and Jönköping Counties, Sweden, February 2020 to February 2022 - Simple models.

	**Monitoring by proactive testing (≥1 negative test), OR (95% CI) (*P* value)**	**Positive SARS-COV-2 test (≥1), OR (95% CI) (*P* value)**
**Main exposures**		
Relocated (1, yes; 0, no)	1.37 (1.35–1.39) (<0.001)	0.65 (0.64–0.66) (<0.001)
Substance abuse (1, yes; 0, no)	0.69 (0.66–0.72) (<0.001)	1.35 (1.30–1.39) (<0.001)
Psychotic disorder (1, yes; 0, no)	0.74 (0.70–0.79) (<0.001)	1.35 (1.30–1.41) (<0.001)
**Moderators**		
Sex (1, male; 0, female)	0.80 (0.79–0.81) (<0.001)	0.67 (0.66–0.67) (<0.001)
Age (continuous)	0.98 (0.98–0.98) (<0.001)	0.98 (0.98–0.98) (<0.001)
**Testing intensity indicators**		
Monitoring (≥1 negative test) (1, yes; 0, no)	2.21 (2.19–2.24) (<0.001)	–
Tested positive (≥1) (1, yes; 0, no)	–	2.21 (2.19–2.24) (<0.001)

Odds ratios (simple logistic regression models) represent associations between substance abuse, and psychotic disorder, and likelihood of COVID-19 monitoring (≥1 negative test) and having tested positive for SARS-COV-2 (≥1 positive test). The likelihood of COVID-19 monitoring was corrected for having tested positive for SARS-COV-2 and vice versa. *P* values >0.05 are not significant.

Table S6. Likelihood of Hospitalization With COVID-19 among Adult Residents and Psychiatric Populations in Östergötland and Jönköping Counties, Sweden, February 2020 to February 2022 – Simple models.

	**Hospitalization pre-vaccination, OR (95% CI) (*P* value)**	**Hospitalization in vaccination period, OR (95% CI) (*P* value)**
**Main exposures**		
Relocated (1, yes; 0, no)	2.12 (1.96–2.31) (<0.001)	2.08 (1.93–2.24) (<0.001)
Substance abuse (1, yes; 0, no)	1.78 (1.47–2.16) (<0.001)	1.96 (1.65–2.33) (<0.001)
Psychotic disorder (1, yes; 0, no)	2.47 (1.99–3.06) (<0.001)	2.17 (1.75–2.68) (<0.001)
**Moderators**		
Sex (1, male; 0, female)	1.13 (1.05–1.22) (0.001)	1.19 (1.12–1.28) (<0.001)
Age (continuous)	1.04 (1.04–1.04) (<0.001)	1.03 (1.02–1.03) (<0.001)

Odds ratios (simple logistic regression models) represent associations between substance abuse, and psychotic disorder and hospitalization with COVID-19 during two periods: pre-vaccination (up to 31 January 2021), and during vaccination (from 1 February to 2021 and up to 15 February 2022). *P* values >0.05 are not significant.

Table S7. Likelihood of Hospitalization with COVID-19 in Vaccination Period in Patients Diagnosed with Substance Abuse or a Psychotic Disorder Displayed by Vaccination Status – Simple models.

	**Relocated status (*n* = 110,451), OR (95% CI) (*P* value)**	**Substance abuse diagnosis (*n* = 14,262), OR (95% CI) (*P* value)**	**Psychotic disorder (*n* = 8387), OR (95% CI) (*P* value)**
**Main exposure**			
Remaining unvaccinated (1, yes; 0, no)	1.13 (0.99–1.29) (0.063)	1.53 (1.08–2.18) (0.018)	1.50 (0.97–2.34) (0.071)
**Moderators, relocated status, and psychiatric comorbidity**			
Relocated (1, yes; 0, no)	–	1.57 (0.95–2.59) (0.076)	1.78 (1.08–2.93) (0.025)
Substance abuse (1, yes; 0, no)	1.64 (1.02–2.62) (0.039)	–	1.58 (0.97–2.57) (0.064)
Psychotic disorder (1, yes; 0, no)	1.96 (1.25–3.06) (0.003)	1.72 (1.09–2.72) (0.021)	–
Sex (1, male; 0, female)	1.20 (1.06–1.37) (0.005)	1.15 (0.79–1.67) (0.461)	1.17 (0.77–1.78) (0.465)
Age (continuous)	1.04 (1.04–1.05) (<0.001)	1.02 (1.01–1.03) (<0.001)	1.01 (1.00–1.02) (0.213)

Odds ratios (simple logistic regression models) representing associations between vaccination status and hospitalization with COVID-19 among substance abuse patients and patients with psychotic disorders when vaccination was administrated (after 31 January 2021). *P* values >0.05 are not significant.
